# Supplementary material for: Complexome profiling of the Chlamydomonas psb28 mutant reveals TEF5 as an early PSII assembly factor
Source: Plant Cell. 2025 Mar 17;37(6):koaf055. doi: 10.1093/plcell/koaf055 (PMC12164590; doi:10.1093/plcell/koaf055)
Supplement: koaf055_Supplementary_Data [file koaf055_supplementary_data.zip › psb28_interactive.zip!/plots/intensity/qualityPlots/peptides/_Cre16.g658526.t1.1.html]

### Cre16.g658526.t1.1

Trivial name:   
SeqCoverage [median]: 11.20  
Mass: 73.99 kDa  
  
 

| Sequence | MissedCleavage | Mass [Da] | wtIntensity | muIntensity | Score |
| --- | --- | --- | --- | --- | --- |
| EFDTTDEPIVR | 0 | 1320.62 | 1077.37 | 1899.77 | 117.01 |
| ELVADSLGK | 0 | 930.50 | 0.00 | 816.54 | 61.96 |
| GLLSDDNESESHLR | 0 | 1570.72 | 0.00 | 2617.43 | 53.97 |
| GVEDLYPAELSGGMK | 0 | 1564.74 | 1010.17 | 1122.37 | 80.98 |
| LAAGLLQPDK | 0 | 1024.59 | 411.01 | 1141.63 | 64.65 |
| LAAGLLQPDKGIVK | 1 | 1421.86 | 1362.42 | 6631.13 | 63.09 |
| RGEAVGIIGASGTGK | 1 | 1371.75 | 1794.13 | 1034.71 | 72.17 |
|  |  |  | 5655.1 | 15263.6 |  |
